# A Molecular and Epidemiological Investigation of a Large SARS-CoV-2 Outbreak in a Long-Term Care Facility in Luxembourg, 2021

**DOI:** 10.3390/geriatrics8010019

**Published:** 2023-01-26

**Authors:** Corinna Ernst, Yolanda Pires-Afonso, Dritan Bejko, Conny Huberty, Thomas G. Dentzer, Anke Wienecke-Baldacchino, Eric Hugoson, Daniel Alvarez, Murielle Weydert, Anne Vergison, Joël Mossong

**Affiliations:** 1 Luxembourg Health Directorate, L-1273 Luxembourg, Luxembourg; 2 Laboratoire National de Santé, L-3583 Dudelange, Luxembourg

**Keywords:** COVID-19, SARS-CoV-2, public health, elderly, nursing home, outbreak, mortality, whole genome sequencing, antibodies, infection control

## Abstract

In spring 2021, a long-term care facility (LTCF) of 154 residents in Luxembourg experienced a large severe, acute respiratory-syndrome coronavirus 2 (SARS-CoV-2) outbreak a few days after a vaccination campaign. We conducted an outbreak investigation and a serosurvey two months after the outbreak, compared attack rates (AR) among residents and staff, and calculated hospitalization and case-fatality rates (CFR). Whole genome sequencing (WGS) was performed to detect variants in available samples and results were compared to genomes published on GISAID. Eighty-four (55%) residents and forty-five (26%) staff members tested positive for SARS-CoV-2; eighteen (21%) residents and one (2.2%) staff member were hospitalized, and twenty-three (CFR: 27%) residents died. Twenty-seven (21% of cases) experienced a reinfection. Sequencing identified seventy-seven cases (97% of sequenced cases) with B.1.1.420 and two cases among staff with B.1.351. The outbreak strain B.1.1.420 formed a separate cluster from cases from other European countries. Convalescent and vaccinated residents had higher anti-SARS-CoV-2 IgG antibody concentrations than vaccinated residents without infection (98% vs. 52%, respectively, with >120 RU/mL, *p* < 0.001). We documented an extensive outbreak of SARS-CoV-2 in an LTCF due to the presence of a specific variant leading to high CFR. Infection in vaccinated residents increased antibody responses. A single vaccine dose was insufficient to mitigate the outbreak.

## 1. Introduction

Before the introduction of a vaccine, COVID-19 took a high toll on the elderly, particularly those living in long-term care facilities (LTCFs). They were at higher risk for severe courses of disease due to not only their age but also due to prevalent underlying medical conditions [1]. Outbreaks in LTCFs were common and associated with high mortality. Living in a congregate setting where transmission is fast and compliance with hygiene rules is challenging increases the risk of infection. Reports of outbreaks in these institutions showed high mortality rates for LTCF residents, often exceeding 25% [2,3,4,5].

The emergence of variants of concern (VOCs) in late 2020 posed new challenges for public health authorities and all actors in the healthcare sector. In Luxembourg, the sequencing capacity increased substantially when the Alpha VOC emerged in December 2020. In early February 2021, before this particular outbreak, 26.5% of all cases were sequenced, the majority of which were identified as Alpha (77%), followed by the Beta variant (22.4%) [6].

The European Medicines Agency (EMA) authorized the first COVID-19 vaccines in December 2020 and vaccination was rolled out in Luxembourg by the end of December 2020. Similar to other European countries, LTCF residents and healthcare workers were among the first priority group accordingly vaccinated in phase 1, while vaccine supply was limited [7,8]. Residents were exclusively administered Comirnaty, while 67.1% of staff members working in the healthcare sector received Vaxzevria and 32.9% received Comirnaty. By the onset of this LTCF outbreak in February 2021, vaccination had just begun: 2.6% of Luxembourg’s population aged 18 years and older had received a first dose, and 0.7% had completed a primary course. Among individuals aged 60 years and older, 3.7% were vaccinated with one dose and 1.7% had completed a primary course [9].

Monthly SARS-CoV-2 screening campaigns in long-term care facilities were introduced at the national level in 2020 within the framework of a national large-scale testing (LST) program. Citizens and cross-border healthcare workers, in particular, were invited by mail to conduct PCR tests at dedicated testing stations, resulting in about 10% of the country’s population being tested every week. Additionally, testing teams performed onsite screening campaigns at LTCFs [10].

On 17 February 2021, the Health Directorate received the first positive SARS-CoV-2 PCR result for a nurse working at a particular LTCF facility. One day later, the first positive result for a resident was received and treated by the contact-tracing team at the Health Directorate. The Health Directorate contacted the LTCF right after detection of the first SARS-CoV-2 positive resident and worked in close collaboration with the LTCF management throughout this outbreak.

We performed an epidemiological analysis of that outbreak by describing its evolution, the variants detected by whole genome sequencing (WGS), and infection control measures. Further, we conducted a serosurvey two months after the outbreak to investigate the association of vaccination and prior infection with anti-SARS-CoV-2 IgG antibodies.

## 2. Materials and Methods

### 2.1. Data Source

The present observational study was based on surveillance data from mandatory electronic reporting on SARS-CoV-2 from all laboratories in Luxembourg and specific case-reporting from LTCFs for residents and staff. Luxembourg’s Health Directorate administered this data and complemented it through routine interviews conducted by a designated contact-tracing team. Each case was called and asked about symptoms, possible incidents of exposure, and their close contacts in the form of a structured interview. As it was generally not possible to interview LTCF residents, information on these cases was provided by LTCF management. Data was collected and processed within the provisions of the national COVID-19 law [11].

### 2.2. Setting and Testing

The LTCF was an integrated care center with 154 single rooms for the elderly with widely differing needs regarding care, ranging from residents with little need for assistance to people with dementia and those who required extensive care. All residents and staff members (including external workers) during the period of outbreak were included in the analysis. Cases included in the study were defined as those having received a positive PCR result for SARS-CoV-2 either from oro- or naso-pharyngeal swabs between 17 February 2021 and 23 March 2021. Subjects were tested either on the basis of having symptoms or in the context of monthly or reactive screening campaigns. Cases with a documented positive PCR result dating more than 60 days ago were considered to be reinfected. Additional data on residents and staff members were provided by the LTCF management.

The first cases were tested after developing symptoms and being present at the LTCF at the time of their pre-symptomatic infectious episode. Therefore, in addition to testing every symptomatic person and their contacts with a high risk of infection, a comprehensive testing of all staff members and residents was also organized on 23 February. In total, four comprehensive molecular screenings were performed during the outbreak. Two screenings were organized by the LTCF management on 23 February and 10 March, and two screenings were organized by the national LST program and took place on 1 March and 15 March 2021.

### 2.3. Sequencing

All positive samples analyzed by laboratories other than the Laboratoire National de Santé (LNS) were sent to the LNS as the national reference laboratory for potential further analysis, i.e., whole genome sequencing. Samples containing at least one CT value below 35 were eligible to be sequenced. Sequencing was performed using an amplicon approach, based on an in-house primer scheme. The primer scheme results in 51 overlapping amplicons with an approximate length of 900 bp and an overlap of 200 bp. Library preparation was done using the ILLUMINA DNA prep kit, following the manufacturers’ instructions. Per run, 192 samples were sequenced according to a 151 bp-paired end approach and indexed with the IDT-ILMN Nextera DNA UD Indexes 384—Nextera DNA Flex set on an Illumina instrument (either MiniSeq^®^ or MiSeqDX^®^ (San Diego, CA, USA)).

Pre-analytical steps are represented by quality control comprising initial-quality inspection (Fastqc), deduplication of paired-end reads, dehumanization by mapping versus human genome (hg20), primer-sequence removal from read extremities (bamclipper in combination with an inhouse python script), and finally, quality trimming (trim_galore, fastqc, -q 20). QCed data have been mapped to NC_045512.1 (SARS-CoV-2 RefSeq sequence of Wuhan-1) with bowtie2.

Subsequent reference mapping-based consensus-sequence generation was done using samtools mpileup (−aa −A −d 0 −B −Q 0) in combination with iVar consenus with a strict parameter -t 0.5 and a minimum depth-to-call-consensus of 20. Variant calling was done using samtools mpileup (−aa −A −d 0 −B −Q 0) in combination with iVar variants with a minimum read depth of 20 and a default minimum frequency threshold of 0.03 to call variants. Lineage assignments and variant annotation were performed by pangolin and VEP, respectively.

### 2.4. Phylogeny

A phylogeny was constructed for samples with at least 95% sequencing coverage with a backbone of B.1.1.420 SARS-CoV-2 sequences from European countries (Italy, Spain, France, Germany, and other European countries grouped) from the first to the last sequencing result (October 2020–May 2021) of the variant uploaded to GISAID [12,13,14]. Multisequence alignment was performed by mapping against the Wuhan-Hu-1 reference genome (NCBI Reference Sequence: NC_045512.2) using minimap2 [15] on the publicly available Pathogenwatch platform (version 19.3.0, https://pathogen.watch/, accessed on 12 December 2022). The resulting phylogenetic tree was downloaded in newick format, annotated, and then visualized on the web application, Interactive Tree of Life (iTOL v6.6) [16]. To reduce the large size of the dataset, singletons were manually excluded, and Luxembourg samples were kept at an 80/20 (resident/staff) ratio based on the total number of samples sequenced. Genome data generated from the LTCF was deposited in GISAID (https://www.gisaid.org/, accessed on 12 December 2022). GISAID accession IDs can be found in Table S1.

### 2.5. Serosurvey

A seroprevalence survey based upon voluntary participation was conducted among LTCF residents on 11 May 2021, i.e., 7 weeks after the last outbreak case. The participants’ dried blood spot sample from a finger prick was analyzed using the Anti-SARS-CoV-2 QuantiVac ELISA (IgG) assay (Euroimmun, Lübeck, Germany) according to the manufacturer’s instructions. Results were categorized as negative (<11 RU/mL), intermediate positive (11–120 RU/mL), or strongly positive (>120 RU/mL).

### 2.6. Statistical Analyzes

The statistical analysis was performed using SPSS Version 27 (Armonk, NY, USA) [17]. The characteristics of residents and staff were described using median and interquartile range for continuous variables and proportions for categorical variables. We calculated the attack rate (AR; number of infected cases divided by the people at risk) to assess the risk of becoming a case, before and during the February outbreak. We estimated 8-day hospitalization and 28-day-case fatality rate (CFR) for residents. Non-parametric Man Whitney-U-test was used to assess associations between infection, hospitalization, or death with age. Pearson’s Chi-Square test was performed to assess associations with sex.

## 3. Results

### 3.1. Demographic and Epidemiological Characteristics

Prior to February 2021, 38 residents and 29 staff members of the LTCF tested positive for SARS-CoV-2. The majority of these cases occurred in two smaller clusters in September 2020 (6 residents, 5 staff members) and between October and December 2020 (30 residents, 20 staff members)—corresponding to 24.7% of total residents and 16.8% of total staff members, which were positive before the February outbreak. In an LST campaign on 14 February, 3 days before the first positive case occurred in the spring outbreak, 139 persons (residents and staff) were tested, and only negative test results were obtained.

Between 17 February and 23 March 2021, a total of 84 and 45 cases of COVID-19 were observed among 154 residents and 173 members of staff, yielding a significantly higher (*p* < 0.001) attack rate among residents (54.5%) compared to that of the staff (26%). During the outbreak, 18 (21.4%) residents were hospitalized and 23 died (CFR 27.4%) in the 28 days following their positive test result (Table 1). Out of the 23 deceased, 12 died in hospital and 11 in the LTCF. The average duration between the date of test result and the date of hospitalization was 4.5 days (SD 10.4). The average duration between the date of test result and the date of death was 8.8 days (SD 4.7). One staff member was hospitalized on 26 February, for which we lack information on the length of stay. The average duration of hospital stay for residents was 4.7 days (SD 3.8).

The hospitalized (median age 89.5) and deceased resident cases (median age 89) appeared to be older than the cases (median age 88) and non-cases (median age 85), although the differences were not significant (*p* > 0.05). There was no significant association between sex and infection, hospitalization, and death in bivariate analysis.

### 3.2. Chronological Description of the Outbreak

**Figure 1:**
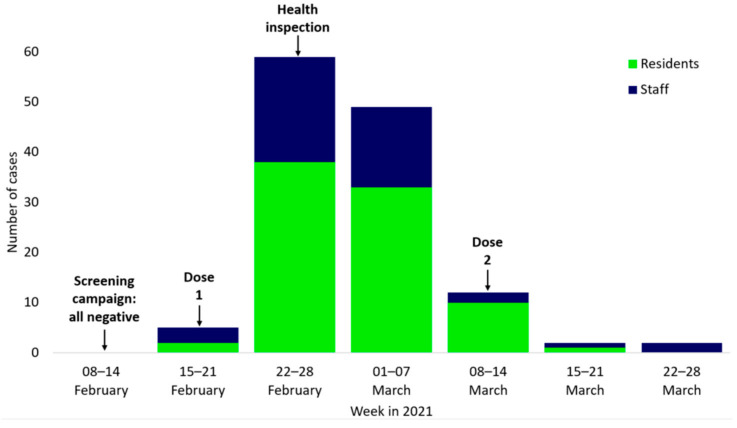
Number of cases between residents and staff during a long-term care facility SARS-CoV-2 outbreak in February–March 2021 in Luxembourg. Screening campaign: all negative: last screening at the LTCF with negative results only; Dose 1: First in-house vaccination campaign; Dose 2: Second in-house vaccination campaign; Health inspection: Health Directorate team visited the LTCF to assess local circumstances and needs.

The epidemic curve of the outbreak is shown in Figure 1.

On 17 February, one resident and one nurse tested positive on the same day. The nurse was symptomatic on 14 February and worked until 15 February. The first detected resident case was a 95-year-old woman who showed symptoms on 17 February and who had a prior SARS-CoV-2 infection three months earlier. Both cases were unvaccinated at the time. One day later, on 18 February, a vaccination campaign was conducted in-house where 142 (92.2%) residents and 79 (45.7%) staff members received their first dose of the SARS-CoV-2 vaccine.

Throughout the following 34 days, despite strengthened infection control measures being implemented, 127 individuals (83 residents, 44 staff members) tested positive. Most (56.6%) of the 129 total cases were detected during two screening campaigns conducted on 23 February and 3 March. Two positive cases among staff were of particular interest from the epidemiological investigation. First there was an internal staff nurse who had administered vaccines to residents and staff members (wearing personal protective equipment); she started having symptoms the day after the campaign (19 February) and tested positive on 21 February. Secondly, there was an external staff member, who was only present in the building on the day of the vaccination campaign (18 February). This external staff member became symptomatic and tested positive five days later on 23 February. It happened that he was one of the 29 cases out of 39 individuals who tested positive on 23 February and was identified with the variant B.1.1.420. The last cases were detected in two staff members on 23 March. On 7 April, all surviving and convalescent residents and staff members tested negative during a screening campaign.

### 3.3. Variants

Of the one hundred and twenty-nine detected cases, seventy-nine (61%) were successfully sequenced. Sixty-one (73%) residents and sixteen (36%) staff members were infected with the dominant outbreak variant (B.1.1.420). As expected, there was little variation within the outbreak isolates. Interestingly, the Beta variant (B.1.351) was detected in samples from two staff members but not in residents. These two sequences belonging to Beta variant suggested that these two infections were acquired independently.

**Figure 2:**
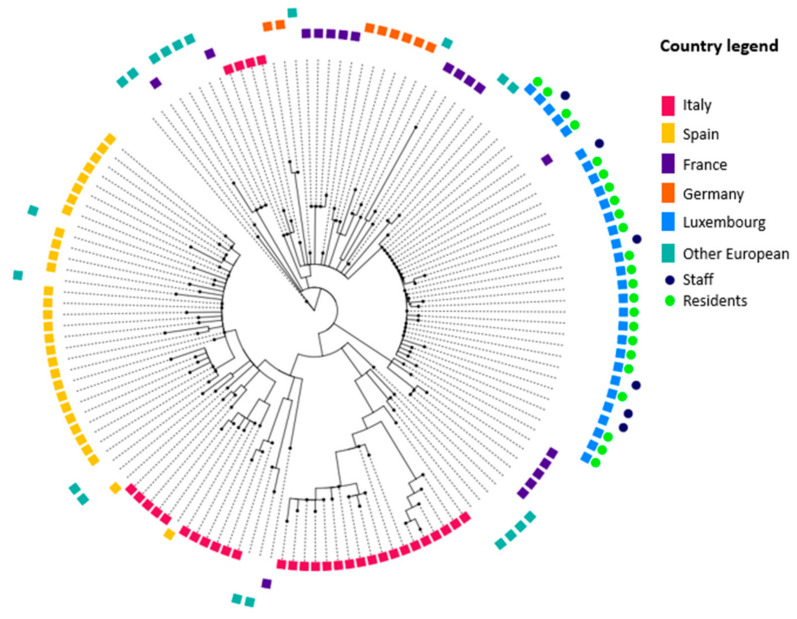
Phylogenetic tree of B.1.1.420 cases comparing resident and staff status during a long-term care facility SARS-CoV-2 outbreak in February–March 2021 in Luxembourg with reference sequences from other European countries.

Comparison of the B.1.1.420 sequences with the reference results from other European countries (Italy, Spain, France, Germany, other European countries grouped) shows distinct genetic characteristics of the main outbreak variant (Figure 2). Genomes submitted from Luxembourg represented 11.4% of all B.1.1.420 genomes on GISAID. One case from France (sampling date: 30 March 2021) shared similar genetic characteristics with the outbreak strain and appears as part of the LTCF cluster on the phylogenetic tree.

### 3.4. Reinfections

Eighteen residents and nine staff members tested positive for the second time in this outbreak. Most (85%) reinfected cases showed symptoms during their second infection, while only 22% had reported symptoms during their previous infection. Five (19%) cases reported symptoms during both infections. The average time between infections was 138 days (SD 74.4) days. Twenty of twenty-seven cases had tested negative between their first and second positive test during the spring outbreak. Three residents were hospitalized, and five died within 3–16 days after their second positive result. Eighty-three percent of reinfections among residents were sequenced as belonging to the main outbreak variant B.1.1.420. Previous infection did not appear to protect against infection, hospitalization, or death during this outbreak.

### 3.5. Seroprevalence

A seroprevalence survey was conducted among residents in May 2021, 7 weeks after the outbreak. Of the total 154 residents, 73 (47.4%) participated in the survey. By then, all participants (100%) had received two doses of Comirnaty vaccine. None (0%) had a negative result, 13 (17.8%) had an intermediate positive result (11–120 RU/mL) and 60 (82.2%) had a strongly positive anti-SARS-CoV-2 IgG result (>120 AU/mL). Residents with prior infection had higher antibody levels: 47 of 48 residents (97.9%) who had been prior COVID-19 cases had a strongly positive anti-SARS-CoV-2 IgG result compared to 13 out of 25 residents (52.0%) who had not been prior COVID-19 cases (*p* < 0.001).

### 3.6. Outbreak Control Measures

The Health Directorate was in regular contact with the LTCF management during this outbreak from 22 February until 4 April 2021. Tracing did not allow for the identification of a single source for all cases, and it remains unclear which factors contributed the most to the spread. Many residents gathered together for the vaccination session in the same waiting area, with poorly organized circulation and physical distancing. While mask wearing was mandatory, not all residents wore them consistently.

Before the outbreak, residents and staff members were recommended to wear masks, keep physical distance, and practice good hand hygiene. After the detection of the first few cases in February, staff members were asked to wear FFP2 masks and blouses. Residents were also asked to wear masks. Additional measures were implemented on top of contact and droplet precautions and the isolation of positive cases and close contacts, such as: The closing of restaurants from 21 February onwards—meals were served in the residents’ rooms. A visitor stop was implemented, except for residents at the end of life. Additional staff was made available from other facilities belonging to the same company. Oxygen generators for the treatment of residents were available in-house. Given the large extent of the outbreak, the particular variants, and the vulnerable population, the duration of isolation for residents was increased from 10 days to 14 days with a test at the end of the period. Administration of the second dose of vaccine (Figure 1) was scheduled either on 17 March or 31 March. The Health Directorate visited the LTCF on 5 March (Figure 1) to assess the local circumstances and needs. At the beginning of the pandemic, the Health Directorate offered training sessions and visits to review infection control measures and the organization of cohorts according to local specificities. In total, 41 out of 52 LTCFs were visited, but this particular LTCF was not visited as the LTCF group only received visits in LTCFs that had positive SARS-CoV-2 cases at that time. The cohorting of residents was not implemented for logistical reasons. Regular testing was coordinated in the context of the national LST program, and screenings were organized by the LTCF. New clients were accepted from hospitals, even those with a positive SARS-CoV-2 test.

As the outbreak raised public interest, the national parliament assigned an independent working group to analyze the clusters in LTCFs, from which they provided recommendations including setting up a national multi-sectoral working group for monitoring and managing COVID-19 infections in LTCFs [10].

## 4. Discussion

This retrospective analysis describes a large LTCF outbreak with 84 residents and 45 staff members testing positive for SARS-CoV-2 within 35 days between February and March 2021. The results indicate that large and severe SARS-CoV-2 outbreaks still occurred one year after the pandemic’s onset, despite prior outbreak incidents. LTCF residents were recognized as a “medically and socially vulnerable group” [18]. As described previously [19,20], residents appeared to be more susceptible to infection, hospitalization, and death than staff members. Similarly, these outbreaks started with staff members testing positive. Similar to other LTCFs in different countries [19,20,21], this LTCF in Luxembourg had a major SARS-CoV-2 outbreak in 2021. With an attack rate (AR) of 54.5% among residents and 26% among staff, this was a comparatively large outbreak.

Three elements indicate that a certain amount of viral transmission occurred on the day of the in-house vaccination: the timing of the peak of positive SARS-CoV-2 test results, the presence of a likely infectious but pre-symptomatic staff member, and the infection of an external staff member who was most likely only exposed to the outbreak variant on the day of the vaccination campaign.

Studies on Comirnaty’s efficacy imply that the protection begins to take effect 12 days after the first dose, with an efficacy of around 50% between both doses [22,23]. Both studies did not find differences based on potential influencing factors such as age, sex, or ethnicity. Krutikov et al. found a vaccine effectiveness of 16.3% within the 27 days following the first dose, which increased to 51.6% from day 28 onward [24]. These results are interesting in light of the timing of the outbreak in the LTCF in Luxembourg. Our study seems to confirm the low vaccine effectiveness shortly after the first dose. We observed that all infections among residents occurred within the first 27 days after the administration of the first dose. Congregate settings and large events in such can become critical—the in-house vaccination campaign could have been a decisive event for the onset of this outbreak.

During the outbreak, two variants were introduced into the facility. Only one specific variant, B.1.1.420, led to a sustained chain of transmission. This variant was detected sporadically during the same time period in the general population as the LTCF in the same geographic area. Moreover, an analysis of wastewater confirms the observation of the local accumulation of the B.1.1.420 variant [25]. By the time of the outbreak’s onset, the dominant variants circulating in Luxembourg’s LTCFs were the Alpha and Beta variants [10]. Interestingly, one case sequenced in France appeared to be part of the outbreak cluster on the phylogenetic tree. Considering that a part of the staff working at the LTCF consists of cross-border workers living in France and working in Luxembourg, it is possible that this sequencing result identifies an individual working at this particular LTCF.

Globally, B.1.1.420 was detected in 25 countries between August 2020 and September 2021 [26]. Although the first uploaded sequences of B.1.1.420 in GISAID stem from Senegal (Figure S1; Senegal grouped with other African countries) [26], it remains uncertain, if the variant originated in Africa or in Europe [27]. As described by Perez et al., the variant appears to have gained genetic diversity and transmissibility in Senegal and then spread across European countries, i.e., UK, Germany, and France [27,28]. In Luxembourg, the variant B.1.1.420 was found in 143 samples between December 2020 and April 2021 including another small cluster of eleven cases in the same area in another LTCF [26]. The variant B.1.1.420 was characterized by three distinctive mutations of interest in the spike gene (L18F, N440K, D614G), which were also observed in other VOCs [29]. Amino acid substitutions D614G and L18F were detected in both Beta and Gamma VOCs. The spike mutation D614G also occurred in Alpha, Delta, and Omicron VOCs [30]. The aspartic-acid-to-glycine substitution on position 614 (D614G) appears to have an advantage in terms of transmissibility and infectivity [31], and decreased antibody neutralization is linked to the L18F mutation [32]. B.1.1.420 disappeared relatively quickly as Alpha and Delta variants became dominant throughout the year 2021. It remains unclear why the variant has spread widely in the local outbreak, but not beyond. The second variant identified in this outbreak, the Beta variant, was exclusively detected in staff members and did not lead to any transmission chains.

Reinfection can be a common phenomenon in LTCFs, as suggested by the relatively high share of second positive cases after a longer period of time within which there were many negative PCR results. Twenty-seven individuals were reinfected during the February 2021 outbreak. Most residents were asymptomatic during their first infection, while 85% developed symptoms during their second infection. This is in line with observations made in an LTCF outbreak in Kentucky, where five residents tested positive during two outbreaks three months apart and presented more severe disease symptoms during this second infection compared to the first [33]. It is unknown to what extent the virus variant causing the infection influenced the clinical course of disease.

Similar to other findings [34], our results show that while all fully vaccinated LTCF residents had a detectable IgG antibody level, those with prior SARS-CoV-2 infection had higher IgG antibody titers than those without prior infection. Whether facilities that have experienced no or a few cases of COVID-19 are more vulnerable (at comparable vaccine coverage) to future outbreaks than those that had many cases remains an open question.

Other articles on SARS-CoV-2 cases in LTCFs suggest an association of outbreaks with the size of the LTCF and being part of a larger LTCF provider. The studied outbreak occurred in a relatively large LTCF, which is among the ten largest integrated centers for the elderly in Luxembourg. Larger facilities and facilities that belong to LTCF groups seem to show more severe outbreaks [35,36,37]. Providers managing numerous houses obtain the opportunity to easily mitigate staff shortages by relocating their workforce, but they face the challenge of an increased risk for virus propagation, particularly if staff cohorting is not implemented [38].

Public health measures navigated by the Health Directorate in close collaboration with the LTCF, such as extensive testing, the strict isolation of positive cases and high risk contacts, stopping outside visits, and closing of restaurants have likely limited the extent of the outbreak. Cohorting may be an effective measure in case of outbreaks in LTCFs [38,39], but it could not be implemented here as LTCF management, the residents, and their families are often reluctant to move residents from their rooms, which is considered their home. Cohorting in combination with a comprehensive testing strategy could accelerate the effective containment of LTCF outbreaks in the future [40] as it was the case in most LTCFs in the country.

These findings are subject to the following limitations. First, as residents of LTCFs are usually not contacted directly by the Health Directorate, and symptoms are reported based on the LTCFs management and staff information, data on symptom status might be incomplete. Second, the sequencing information of cases is incomplete, either because samples were not available or because viral load was too low. Further, vaccination data and information on SARS-CoV-2 infection for staff might be incomplete as almost half (47%) of the staff working in Luxembourg’s LTCFs reside outside of Luxembourg and could have received their vaccination or a positive SARS-CoV-2 test result in their country of residence.

Our findings should be taken into account when considering how to roll out any event, i.e., future in-house vaccination campaigns and testing in LTCFs. Regular testing for all staff and residents should be performed, irrespective of prior occurrence of outbreaks and also for those who have previously tested positive. One question that will remain open is whether cohorting residents and staff members could have further limited the size of this outbreak.

## 5. Conclusions

We document an extensive outbreak of SARS-CoV-2 in an LTCF with a specific variant leading to high CFR. By analyzing the evolution and different influential factors of this outbreak, we identified several contributing elements. The outbreak peaked one week after the first vaccination campaign in this LTCF, implying that the event played a role in the virus propagation. The findings underscore that the rollout of any event, such as vaccination campaigns, should be organized under rigorous precaution, and further, that testing is important to identify the magnitude of an outbreak and could become even more effective when combined with cohorting.

Whole genome sequencing helped us to better understand variant introduction and the respective evolvement of an outbreak, as seen here with the special variant B.1.1.420. It remains unclear why this variant spread so extensively in the LTCF despite its low prevalence in the general population and the fact that some residents and staff already had experienced an infection and/or one dose of a two-dose vaccine schema. A single vaccine dose was insufficient to mitigate the outbreak. Serological data suggests that residents with prior infection and full vaccination could be better protected than those without evidence of prior SARS-CoV-2 infection and full vaccination only.

## Figures and Tables

**Table 1:** Characteristics of COVID-19 cases at the LTCF.

	Population	Cases before Spring Outbreak	Cases during Spring Outbreak	Hospitalized Spring Outbreak	Deaths Spring Outbreak
	N	N (AR ^1^)	N (AR)	N (HR ^2^)	N (CFR ^3^)
Residents	154	38 (24.7%)	84 (54.5%)	18 (21.4%)	23 (27.4%)
Female	110	25 (22.7%)	61 (55.5%)	11 (18%)	15 (24.6%)
Male	44	13 (29.5%)	23 (52.3%)	7 (30.4%)	8 (34.8%)
Median age (IQR ^4^)	87 (81–91)	88 (82–93)	88 (83–91)	89.5 (82–90)	89 (82–91)
Staff	173	29 (16.8%)	45 (26%)	1 (2.2%)	0 (%)
Female	139	21 (15.1%)	39 (28.1%)	1 (2.6%)	0 (%)
Male	34	8 (23.5%)	6 (17.6%)	0 (%)	0 (%)
Median age (IQR)	40 (33–51)	40 (32–50)	39 (32–48)	43	NA
Total	327	67 (20.5%)	129 (39.4%)	19 (14.7%)	23 (17.8%)

^1^ AR = attack rate; ^2^ HR = hospitalization rate; ^3^ CFR = case fatality rate; ^4^ IQR = interquartile range.

## Data Availability

Accession IDs of sequencing results deposited in GISAID can be found in Supplementary Materials (Table S1).

## Supplementary Materials

The following supporting information can be downloaded at: https://www.mdpi.com/article/10.3390/geriatrics8010019/s1, Figure S1: B.1.1.420 variant by country or region per month. Table S1: accession IDs of genome data deposited in GISAID.
