# Supplementary material for: A Molecular and Epidemiological Investigation of a Large SARS-CoV-2 Outbreak in a Long-Term Care Facility in Luxembourg, 2021
Source: Geriatrics (Basel). 2023 Jan 26;8(1):19. doi: 10.3390/geriatrics8010019 (PMC9957261; doi:10.3390/geriatrics8010019)
Supplement: Supplementary file 1 [file geriatrics-08-00019-s001.zip › Table S1. GISAID accession ID.pdf]

Table S1: GISAID accession IDs.

Latest PangolinLineage	GISAID Accession ID
B.1.1.420	EPI_ISL_1383943
B.1.1.420	EPI_ISL_1383952
B.1.1.420	EPI_ISL_1383961
B.1.1.420	EPI_ISL_1383972
B.1.1.420	EPI_ISL_1383976
B.1.1.420	EPI_ISL_1383993
B.1.1.420	EPI_ISL_1384003
B.1.1.420	EPI_ISL_1384470
B.1.1.420	EPI_ISL_1384517
B.1.1.420	EPI_ISL_1384520
B.1.1.420	EPI_ISL_1384529
B.1.1.420	EPI_ISL_1384548
B.1.1.420	EPI_ISL_1384549
B.1.1.420	EPI_ISL_1384553
B.1.1.420	EPI_ISL_1384615
B.1.1.420	EPI_ISL_1384616
B.1.1.420	EPI_ISL_1384617
B.1.1.420	EPI_ISL_1384618
B.1.1.420	EPI_ISL_1384619
B.1.1.420	EPI_ISL_1384620
B.1.1.420	EPI_ISL_1384621
B.1.1.420	EPI_ISL_1384622
B.1.1.420	EPI_ISL_1384623
B.1.1.420	EPI_ISL_1384624
B.1.1.420	EPI_ISL_1384625
B.1.1.420	EPI_ISL_1384626
B.1.1.420	EPI_ISL_1384627
B.1.1.420	EPI_ISL_1384629
B.1.1.420	EPI_ISL_1384630
B.1.1.420	EPI_ISL_1384631
B.1.1.420	EPI_ISL_1384632
B.1.1.420	EPI_ISL_1384633
B.1.1.420	EPI_ISL_1384634
B.1.1.420	EPI_ISL_1384635
B.1.1.420	EPI_ISL_1384636
B.1.1.420	EPI_ISL_1498604
B.1.1.420	EPI_ISL_1498628
B.1.1.420	EPI_ISL_1523202
B.1.1.420	EPI_ISL_1916531
B.1.351	EPI_ISL_1917160
B.1.1.420	EPI_ISL_1917161
B.1.1.420	EPI_ISL_1917551
B.1.1.420	EPI_ISL_1917552
B.1.1.420	EPI_ISL_1917553
B.1.1.420	EPI_ISL_1917554

B.1.1.420	EPI_ISL_1917556
B.1.1.420	EPI_ISL_1917557
B.1.1.420	EPI_ISL_1917558
B.1.1.420	EPI_ISL_1917559
B.1.1.420	EPI_ISL_1917561
B.1.1.420	EPI_ISL_1917562
B.1.1.420	EPI_ISL_1917563
B.1.1.420	EPI_ISL_1917564
B.1.1.420	EPI_ISL_1917565
B.1.1.420	EPI_ISL_1917569
B.1.1.420	EPI_ISL_1917570
B.1.1.420	EPI_ISL_1917572
B.1.1.420	EPI_ISL_1917573
B.1.1.420	EPI_ISL_1917574
B.1.1.420	EPI_ISL_1917576
B.1.1.420	EPI_ISL_1917578
B.1.1.420	EPI_ISL_1917579
B.1.351	EPI_ISL_3148326
B.1.1.420	EPI_ISL_1917580
