# Supplementary figures and images for: A Molecular and Epidemiological Investigation of a Large SARS-CoV-2 Outbreak in a Long-Term Care Facility in Luxembourg, 2021
Source: Geriatrics (Basel). 2023 Jan 26;8(1):19. doi: 10.3390/geriatrics8010019 (PMC9957261; doi:10.3390/geriatrics8010019)

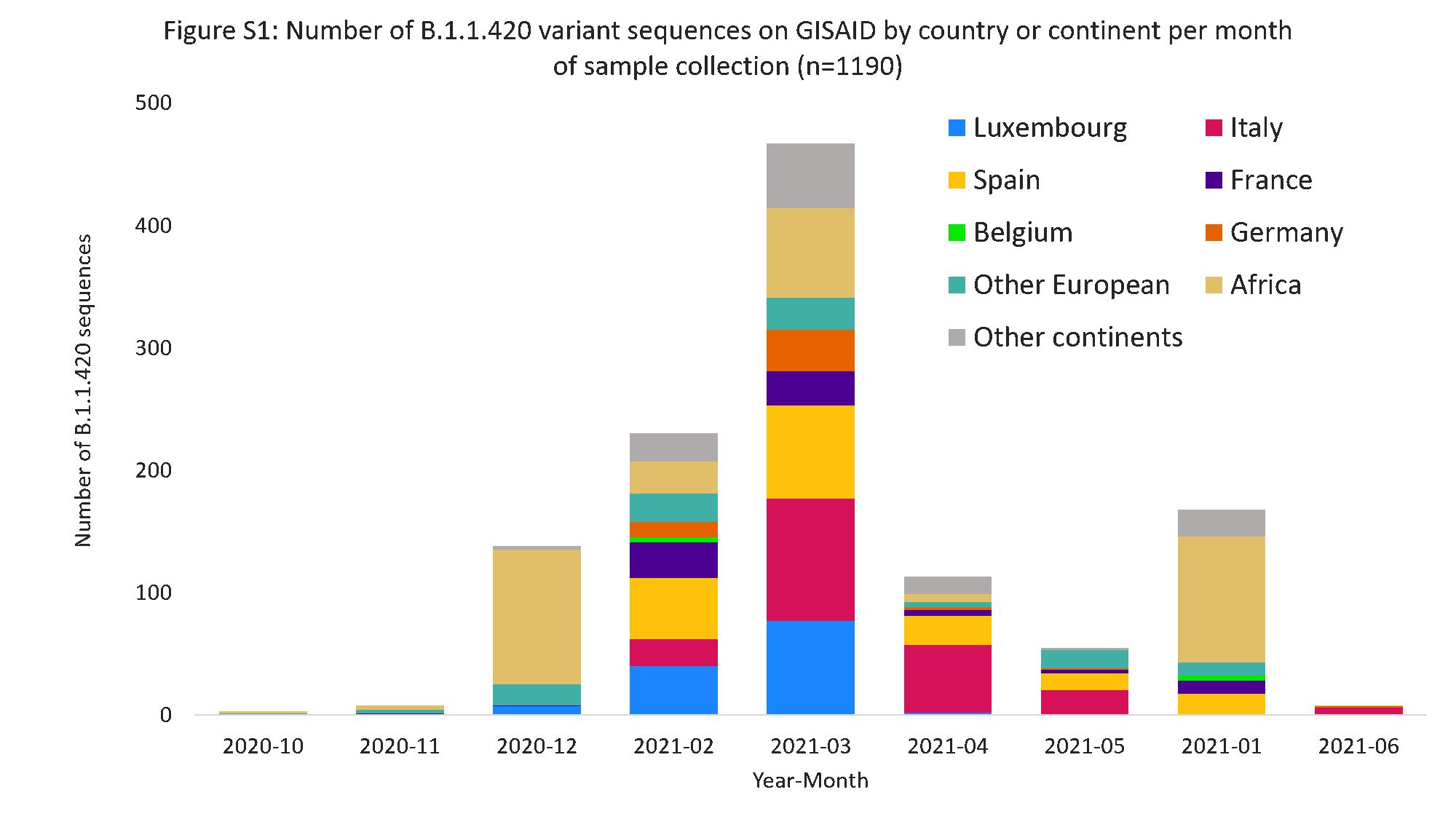

Supplement: Supplementary file 1 [file geriatrics-08-00019-s001.zip › Figure S1. Epi Curve B.1.1.420 by country.png]
